# Present and Future Applications of Artificial Intelligence in Kidney Transplantation

**DOI:** 10.3390/jcm13195939

**Published:** 2024-10-05

**Authors:** Evgenia Kotsifa, Vasileios K. Mavroeidis

**Affiliations:** 1 Second Propaedeutic Department of Surgery, National and Kapodistrian University of Athens, General Hospital of Athens “Laiko”, Agiou Thoma 17, 157 72 Athens, Greece; 2 Department of Transplant Surgery, North Bristol NHS Trust, Southmead Hospital, Bristol BS10 5NB, UK

**Keywords:** renal transplantation, machine learning, deep learning, artificial neural network, support vector machines, surgical data science, computer-aided diagnosis, renal failure, chronic kidney disease, robotics

## Abstract

Artificial intelligence (AI) has a wide and increasing range of applications across various sectors. In medicine, AI has already made an impact in numerous fields, rapidly transforming healthcare delivery through its growing applications in diagnosis, treatment and overall patient care. Equally, AI is swiftly and essentially transforming the landscape of kidney transplantation (KT), offering innovative solutions for longstanding problems that have eluded resolution through traditional approaches outside its spectrum. The purpose of this review is to explore the present and future applications of artificial intelligence in KT, with a focus on pre-transplant evaluation, surgical assistance, outcomes and post-transplant care. We discuss its great potential and the inevitable limitations that accompany these technologies. We conclude that by fostering collaboration between AI technologies and medical practitioners, we can pave the way for a future where advanced, personalised care becomes the standard in KT and beyond.

## 1. Introduction

Artificial intelligence (AI) refers to the simulation of human intelligence in computer systems that are designed to think and mimic the human brain [1]. AI technologies include machine learning, natural language processing, computer vision, robotics and expert systems [2].

Machine learning (ML) is a subfield of AI that focuses on developing algorithms and models that enable computers to learn from and make predictions or decisions based on large amounts of data without explicit programming [3]. There are several types of ML algorithms, categorised into three main groups: supervised learning, unsupervised learning and reinforcement learning. Deep learning (DL) is a subset of ML based on neural networks. DL models are designed to mimic the way the human brain processes information, using multiple layers of nonlinear processing units to provide an output [4].

In supervised learning, the algorithm is trained on a labelled dataset, learning to map input data (independent variables) to the correct output (dependent variables) by finding patterns within this dataset [5]. Two popular examples of supervised learning are artificial neural networks (ANNs) and support vector machines (SVMs). 

AI has a wide range of applications across various sectors, including healthcare, finance, transportation, entertainment and more. In medicine, AI has already made an impact in several fields, rapidly transforming healthcare delivery through its growing applications in diagnosis, treatment and overall patient care. Emerging algorithms have developed to address complex medical problems, filling the void where progress in the medical field has plateaued [6]. Even though AI’s integration into medicine is relatively recent, it is expected to establish substantial advances, exerting notable effects on physicians, patients and healthcare systems [7].

Chronic kidney disease (CKD) represents a major public health problem worldwide, with its global prevalence increasing over the years, estimated in recent years between 8 to 16% [8,9]. Diabetes, hypertension and obesity are prominent factors contributing to CKD incidence. Moreover, in certain areas of the world with pronounced CKD rates, the exact causative factors may still elude identification [10]. Advanced CKD patients face elevated risks of myocardial infarction, stroke and mortality compared to the general population. However, early detection and prompt intervention in CKD can delay the loss of kidney function and reduce the risk of complications such as cardiovascular events [11].

For some patients with end-stage kidney failure (ESKF), kidney transplantation (KT) is the best treatment option, providing better quality of life and longer survival. Nevertheless, for numerous patients, issues relating to fitness and medical eligibility, surgical considerations and the relative scarcity of donor organs, among other factors, may make dialysis the only viable option [12]. 

While KT is generally considered the best treatment option for ESKF, it does come with its own set of challenges and potential risks. These include various surgical complications, such as vascular, urological and wound-related, as well as the risk of graft rejection. Additionally, patients undergoing KT must contend with potential drug interactions, increased susceptibility to infections and several long-term complications (cardiovascular, chronic allograft nephropathy and malignancies related to long-term immunosuppression) [13,14].

**Figure 1:**
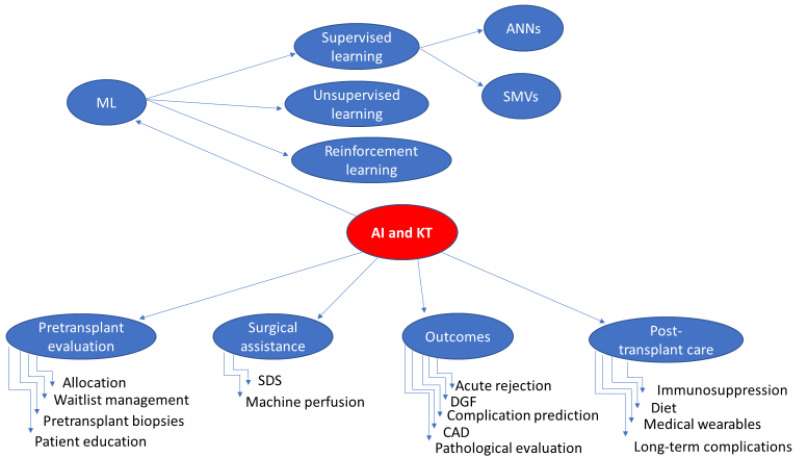
Applications of Artificial Intelligence in Kidney Transplantation. AI: artificial intelligence, KT: kidney transplantation, ML: machine learning, ANNs: artificial neural networks, SVMs: support vector machines, SDS: surgical data science, DGF: delayed graft function, CAD: computer-aided diagnosis.

The purpose of this comprehensive review is to explore the present and future applications of AI in KT (Figure 1). 

## 2. Current and Evolving Applications of AI in KT

### 2.1. Pretransplant Evaluation of Donors/Recipients 

#### 2.1.1. Allocation and Donor-Recipient Matching

The process of transplantation begins with the organ offer, and the decision to accept or reject the offered organ is of high importance. Since not all patients on the waiting list for KT would benefit satisfactorily from all donated kidneys, especially if the graft comes from an expanded criteria donor (ECD) [15], several decision-making tools have been developed to facilitate allocation and reduce discarded organs. The Kidney Donor Risk Index (KDRI), initially developed in 2009, combines 14 donor and transplant factors to predict the risk of graft failure after KT into a single number [16]. The researchers who developed the index concluded that transplants of kidneys with high KDRI (>1.45) are associated with decreased 5-year graft survival compared with the lowest KDRI quintiles. Subsequently, a variant of the KDRI was developed, named the Kidney Donor Profile Index (KDPI) [17]. The latter is based only on donor characteristics and is normalised to a percentile score. Organs with a KDPI ≥ 85%, also known as “high KDPI” organs, are associated with reduced 5-year survival and greater risk of graft failure compared to kidneys with KDPI < 85%. 

To accomplish the best donor-recipient matching, an additional score was developed, namely the Estimated Post-Transplant Survival Score (EPTS). EPTS is a numerical measure combining four recipient parameters (candidate’s age and time on dialysis, current diagnosis of diabetes and prior solid organ transplants) in order to predict post-transplant survival and aid the allocation of donor kidneys [18]. Both KDPI and EPTS were implemented as part of the Kidney Allocation System (KAS) in 2014 by the United Network for Organ Sharing (UNOS) [19]. The current allocation system assigns priority to the top 20% of kidneys (as denoted by KDPI < 20) to patients with an EPTS of ≤20 [20]. Even though the use of the above scores provides an objective tool and their implementation may facilitate the identification of high-risk yet viable kidneys, there is a risk of adverse selection, paradoxically leading to excess organ discards. Bae et al. studied the discard rates before and after the implementation of KDPI. They reported no significant change in discard rate before (18.1%) and after KDPI (18.3%) among the entire population (adjusted odds ratio [aOR] = 1.04 [95% CI 0.97–1.10], *p* = 0.3). Nevertheless, among kidneys in which ECD and KDPI indicators were discordant, standard criteria donor (SCD) kidneys with KDPI > 85 were at increased risk of discard in the KDPI era (aOR = 1.42 [95% CI 1.07–1.89], *p* = 0.02) [21].

As the number of patients on dialysis increases and the number of organ donors remains limited, AI algorithms can be utilised to create a wiser allocation system. As stated already, AI algorithms have the ability to analyse large amounts of data. By considering factors such as blood type, tissue compatibility, donor-recipient age and medical history, AI can help identify the best matches and reduce the risk of rejection [6]. Furthermore, AI algorithms can assist in allocating organs more efficiently by analysing historical data and real-time information. This enables transplant centres to make data-driven decisions on how to allocate organs based on factors such as geographic distribution, transportation logistics and patient demographics [20]. Since the goal of smart donor-recipient matching is to maximise the functional lifespan of the donated organ, multiple studies have used ML algorithms to predict KT outcomes [22,23,24,25,26,27].

Bae et al. developed a prediction tool, available online, that estimates 5-year post-KT survival based on combinations of KDPI and EPTS scores using random forest algorithms. The researchers concluded that their model could support individualised decision-making on kidney offers in clinical practice [23]. In a different study, Brown et al. developed an ML Bayesian Belief Network, a form of graphical model that represents a set of variables and their conditional dependencies, functioning as a pretransplant organ-matching tool. Their model was able to predict graft failure within the first year or three years (sensitivity 40%; specificity 80%; area under the curve, AUC, 0.63) by analysing recipient body mass index (BMI), gender, race and donor age as parameters [24]. 

More recently, researchers developed an ANN model based on data from past organ donors, recipients and transplant outcomes in the United States and compared it with other survival analysis models. On the basis of the C-index, the ANN models had better discriminative ability than the Cox model and random survival forest model (0.650, 0.661 and 0.659 vs. 0.646 and 0.644, respectively). They concluded that their model could effectively predict KT survival and support optimal donor-recipient matching [27]. 

Kilambi et al. developed a decision-tree methodology to calculate the survival benefit of accepting a kidney vs. the survival benefit of rejecting it. Evaluating up to one year of future offers, the tool attains 61% accuracy. This methodology could help form personalised transplant decision-making [25]. 

Mark et al. combined different models to predict the 5-year post-transplant survival of KT recipients. Their proposed ML model, taking into account both donor and recipient characteristics, outperformed the EPTS (concordance index of 0.724 vs. 0.697), concluding that it has the potential to significantly improve the matching of organs to recipients [26].

Finally, Ali et al. developed a risk-stratification index using AI techniques. They used data from 156,749 Deceased-Donor KT between 2007 and 2021 from the UNOS database to train four ML models to predict death-censored graft survival. One of these models showed the best discriminative performance (area under the curve [AUC] = 0.66, 0.67, and 0.68 at 6, 9 and 12 years post-transplant), with time-dependent concordance (CTD) index at 0.66, outperforming the KDPI model (CTD: 0.59 and AUC: 0.60). They concluded that their models could aid decision-making in organ allocation [22]. 

Currently, AI-driven decision-making tools, including those mentioned earlier and others, are not yet fully integrated into formal kidney allocation guidelines. Instead, they are primarily used to supplement rather than replace human judgment.

#### 2.1.2. Waitlist Management 

Since the waiting period for a KT may be prolonged due to the limited availability of donors, it is essential for patients currently on the waiting list to maintain good health while awaiting transplantation. AI can optimise waitlist management by analysing vast amounts of data, including patient demographics, medical history, donor availability and transplant centre statistics to identify patterns and factors that influence waitlist times. Recently, machine learning has been used to develop a prediction model estimating the median waiting time on the list. This model aims to facilitate clinicians regarding management strategies for patients on the list, ultimately contributing to a more efficient use of resources. The researchers considered age, subregion, calculated panel reactive antibody (cPRA) and frequency of human leukocyte antigen (HLA) -DR, -B, -A as major characteristics affecting the time deceased donor recipients spend on a waiting list and developed a prediction model which is available online [28]. Another study for patients on the waitlist used a form of AI to stratify candidates according to their cardiac risk. The researchers utilised clinical risk factors and thallium-201 stress test data to train the algorithm and an ANN, resulting in the development of an expert network that demonstrated a sensitivity of 88%, specificity of 90%, and accuracy of 89% in predicting the 4-year cardiac mortality among KT candidates [29].

#### 2.1.3. Interpretation of Preoperative Grafts Biopsies

As stated before, the shortage of donor kidneys is a long-standing and intractable problem, but it is also exacerbated by a significant discard rate nearing 20% [30]. This high rate of discards is attributed in part to low utilisation rates of pretransplant graft biopsies for pathological assessment. Furthermore, improved electronic accessibility would be essential in addressing this matter [31,32]. Recent studies have demonstrated that AI and DL technologies can improve the effectiveness of pretransplant biopsies [33,34]. Marsh et al. developed a DL model to quantify glomerulosclerosis in kidney biopsy specimens, aiming to decrease the likelihood of unnecessary organ discards. Their model, capable of analysing multiple levels of a section, outperformed the capacity of pathologists, resulting in a 37% lower discard rate [35]. 

In another study, researchers developed a fully automated, DL-based algorithm called RENFAST (Rapid EvaluatioN of Fibrosis And vesselS Thickness) for the segmentation of kidney blood vessels and fibrosis in order to identify vascular and stromal injury of donors’ kidneys [36]. The authors reported that their proposed method showed excellent performance in both blood vessel and fibrosis segmentation (accuracy: 0.8936 and 0.9227, respectively), completing the task in a fraction of the time required by expert pathologists (2 min vs. 20 min per patient). The same authors also presented a second automated algorithm, called RENTAG (Robust EvaluatioN of Tubular Atrophy & Glomerulosclerosis), for the segmentation and classification of glomerular and tubular structures in biopsy specimens of donor kidneys. This algorithm also demonstrated excellent performance (dice score: 0.9529 for glomeruli and dice score: 0.9174 for tubule detection) and, as highlighted by the authors, could serve as a valuable tool to support pathologists’ diagnostic activities [37].

A recent systematic review concluded that existing AI algorithms in pre-implantation kidney biopsy pathology exhibited excellent and promising performance and underlined the significance of expert pathologist annotation to reliably train AI models [38].

#### 2.1.4. Patient Education

Even though AI’s application in patient education is still in its early stages, it has the potential to revolutionise how patients get informed about their health and healthcare. Patient education has always been essential to healthcare, as it empowers patients to understand their medical conditions and engages them in taking active roles in their treatment plans. Well-informed patients tend to adhere more effectively to treatment plans and attain superior health outcomes [39].

Patient educational material is sometimes written at a reading level that exceeds that of many patients. AI technology can be used to rewrite existing patient education materials to suit different levels of reading comprehension [40]. Additionally, AI algorithms can analyse patient data, such as medical history and diagnostic imaging, to generate personalised educational materials tailored to the unique needs and concerns of each patient. This could include information about their specific condition, the transplant process and lifestyle changes they have to adapt to post-transplant.

AI-powered chatbots and virtual assistants can answer common questions, provide information about KT, and offer support throughout the pre- and post-operative periods. These tools can be available 24/7 to address patient queries and alleviate concerns. As of now, AI-powered chatbots are being deployed across various healthcare settings, including weight-loss recommendations and physical activity promotion [39,40,41,42].

### 2.2. Surgical Assistance

#### 2.2.1. AI-Powered Surgical Assistance Systems 

Surgical data science (SDS) is an interdisciplinary field that leverages data science, AI and ML to enhance surgical practice, training and outcomes. The aim is to use advanced computational techniques to improve various aspects of surgery, such as preoperative planning, decision-making and surgical assistance [43]. 

The integration of AI in robotic surgery has resulted in improvements in various parameters such as ergonomics, precision, stability and autonomy. The new AI-driven robots provide real-time feedback and guidance to the surgeon, enhancing the accuracy of procedures. In KT, AI-powered imaging techniques, such as augmented reality (AR), can assist surgeons in optimally visualising the kidney and surrounding structures during transplantation. These technologies can overlay digital images onto the surgical field, offering a detailed, real-time view of the anatomy. The use of three-dimensional AR guidance during KT has the potential to navigate the surgeon with algorithms analysing these images to identify critical structures, suggest optimal incision sites and predict potential complications [44,45]. 

AI technologies can also offer real-time data analysis and decision support. In the operating room, AI systems can continuously monitor the procedures and the patient’s vital signs. Analysing this data in real-time helps predict the remaining duration of the procedure in order to facilitate scheduling or to anticipate the need for resources and detect any anomalies, providing decision support to the surgical team [43]. For example, AI can predict bleeding risks, monitor kidney perfusion and alert the surgeon to potential issues before they become critical.

SDS can also revolutionise surgical training. AI-based simulation platforms can be used to train surgeons in kidney transplant techniques. These platforms provide a realistic and interactive environment where surgeons can ameliorate their skills. Objective Computer-Aided Skill Evaluation (OCASE) of Surgical Technical Skill (OCASE-T) offers an SDS approach that uses computational techniques to assess and quantify the technical skills of surgeons objectively. By incorporating data from various sources, such as motion tracking, video analysis and instrument sensors, it provides a low-cost, high-value feedback evaluation of a trainee’s performance in a systematic and unbiased manner [46].

Finally, AI helps create detailed 3D models of the patient’s anatomy using imaging data. These models can be 3D printed and used for preoperative planning and simulation. Surgeons can practise the procedure on these models, allowing them to anticipate challenges and refine their techniques before performing the actual surgery [47].

#### 2.2.2. Machine Perfusion 

AI also has emerging applications in machine perfusion technology. AI algorithms, particularly ML, can assess the quality of the perfused organs by analysing data collected during machine perfusion, such as flow rates, pressure, oxygen consumption and various blood and urine markers. An experimental study performed on porcine kidneys showed promising results towards establishing this method as an objective quality assessment of donor organs [48]. By analysing all the above data, AI can help predict the viability of the kidneys and identify the organs that are at risk of discard [49]. 

A recent study evaluated the condition of donor kidneys using hyperspectral imaging (HIS) during machine perfusion in combination with ML techniques. HIS is an advanced imaging technique that captures and processes information across a wide spectrum of light, extending beyond the visible range into the infrared and ultraviolet, providing diagnostic information about tissue physiology, morphology and composition [50]. Since HIS produces a vast amount of data, it is usually combined with ML for analysis. The researchers trained various convolutional neural network models to classify the kidneys into three categories: non-functional, limited functional and functional, based on inulin excretion behaviour. The reported accuracy of their model varies from 84 to 96% in the validation set and from 62% to 100% in the test set. Based on their results, they concluded that the combination of HIS and ML algorithms could assess the preoperative kidney quality and support the decision-making process of accepting a potential organ for transplantation [51].

Finally, AI holds an essential place in machine perfusion research. A recent study evaluated the proteomic profile of kidney tissue and urine from machine-perfused discarded organs. Multiple ML algorithms were used to identify the most discriminative proteins during perfusion. The researchers concluded that even brief periods of perfusion induce remarkable metabolic and biochemical changes in marginal organs, suggesting that the proteins modulated by the machine perfusion could be developed as future biomarkers or therapeutic targets [52].

### 2.3. Outcomes 

#### 2.3.1. Earlier Acute Rejection Detection and Diagnosis

Acute rejection (AR) significantly impacts kidney allograft survival. Among different categories of rejection, acute antibody-mediated rejection (aABMR) has been reported in up to 7% of patients, with rates as high as 50% in those with HLA-incompatible transplants [53]. By leveraging AI capabilities, healthcare providers can improve the early detection and diagnosis of AR, ultimately enhancing patient outcomes in organ transplantation.

Fritsche et al. developed an algorithm to detect AR based on the pattern of changes in serum creatinine. Their model was trained using a database of KT patients’ records, and the results were compared against the diagnostic performance of experienced physicians. The model achieved an accuracy of 78 +/– 2%, significantly higher than the performance of physicians (69 +/− 5.3%) (*p* < 0.001). The researchers concluded that this algorithm enables earlier and more accurate diagnosis of critical states [54]. 

Two additional studies assessed the application of AI in AR diagnosis. Metzger et al. used SVM-based classification to develop a diagnostic model for rejection. The algorithm was trained on 39 allograft patients by statistical comparison of capillary electrophoresis mass spectrometry (CE-MS) peptide spectra in urine samples from 16 cases with subclinical acute T-cell-mediated tubulointerstitial rejection and 23 nonrejection controls. The model achieved an AUC value of 0.91, correctly classifying 16 out of 18 subclinical and 10 out of 10 clinical rejections. The study concluded that the set of urinary peptide markers could be used for early diagnosis of AR [55].

Pineda et al. applied ML techniques to identify gene signatures associated with rejection. Their hypothesis was that the use of transcriptome analysis using RNA sequencing (RNAseq) in KT patients’ samples could help identify unique protein-coding and noncoding genes associated with different rejection phenotypes. They evaluated 37 biopsy-paired peripheral blood samples of KT patients with normal/preserved kidney allograft parenchyma, ABMR and T cell-mediated rejection (TCMR) by RNAseq. The study resulted in the identification of 102 genes that perfectly clustered with each rejection phenotype and highly correlated with main histological lesions. Based on their findings, the researchers concluded that the identified critical gene signature found in the blood of KT recipients diagnosed with AR could serve as a potential non-invasive biomarker of the rejection process [56].

#### 2.3.2. Delayed Graft Function Prediction 

Delayed graft function (DGF), defined as the need for dialysis within one week of KT, is associated with increased rates of rejection, short-term morbidity and mortality, hospitalisation and higher costs [57]. Predicting DGF is crucial for improving patient outcomes and managing post-transplant care. AI can assist in DGF prediction through various approaches.

Shoskes et al., in 1998, developed an ANN model trained on retrospective data from 100 cadaveric transplants to predict DGF and validated it with prospective data [58]. The recorded donor variables included age, cause of death, history of hypertension, inotrope use, urine output, and both initial and terminal serum creatinine levels. Recipient factors considered were cold ischemic time, transplant number, antibody sensitisation and HLA match. The model achieved an 80% accuracy rate in predicting DGF. The authors concluded that their model has multiple practical applications, such as aiding in the decision-making process when considering kidneys from marginal donors or high-risk recipients by providing accurate predictions of early function. It can also help organ procurement organisations assess the viability of kidneys from marginal donors and determine the cost-effectiveness of procurement. Additionally, the model can guide transplant centres in tailoring immunosuppressive therapies based on the predicted likelihood of delayed graft function (DGF), thereby optimising treatment and reducing costs. The use of such a model could assist decision-making regarding organ allocation, immunosuppression administration and cost reduction.

Santori et al. proposed a neural network model to predict a delayed decrease in serum creatinine among paediatric kidney recipients. The neural network was trained on a dataset of 107 paediatric kidney recipients using 20 input variables and assuming, for the output variable, the time after three days to reach a serum creatinine level 50% below that before KT. The model was validated with a second set of 41 KT recipients. The overall accuracies of the neural network for the training set, the validation set and the whole patient cohort were 89.1%, 76.92% and 87.14%, respectively. This model could assist in predicting delayed recovery of graft function among paediatric recipients [59].

Kawakita et al. used five ML algorithms (logistic regression, elastic net, RF, ANN and extreme gradient boosting) to predict DGF, trained on a large database of KT recipients. The ANN demonstrated superior performance compared to the baseline model (ROC-AUC of 0.732 vs. 0.705). The authors concluded that ML technology could serve as a personalised risk quantification for DGF prediction [60].

In the same direction, Brier et al. developed an ANN-based model to predict DGF and compared it with traditional logistic regression models. The model resulted in an accuracy of 63%, sensitivity of 63.5% and specificity of 64.8%. They concluded that logistic regression analysis was more sensitive in predicting no DGF development, while the neural network was more sensitive in predicting DGF development [61]. Other studies have also focused on predicting DGF using AI [62,63].

A different study evaluated the risk factors for DGF using ML methods. They assessed several donor maintenance-related variables and concluded that parameters such as urine output, mean arterial pressure, blood glucose and the administration of high-dose vasopressors were associated with DGF [64]. In the same direction, Quinino et al. developed an ML prediction model to differentiate grafts with excellent immediate function (IGF) from those with DGF. The eXtreme Gradient Boosting model yielded the best predictive performance (AUC 0.78; 95% CI 0.71–0.84; sensitivity 0.64; specificity 0.78). The authors concluded that their model could serve as a decision-making tool to identify patients who would benefit from expensive treatments, such as machine perfusion preservation [65].

#### 2.3.3. Post-Transplant Complication Prediction

AI holds significant potential in enhancing the prediction and management of post-kidney transplant complications. By leveraging vast datasets and sophisticated algorithms, AI can provide valuable insights and support healthcare providers in improving patient outcomes. Two studies implemented AI techniques to assess the risk of developing pneumonia as a post-transplant complication [66,67]. Luo et al. utilised ML algorithms to create a model predicting the risk of severe pneumonia in recipients during the perioperative period. They identified three important parameters as risk factors: preoperative pulmonary lesions, reoperation and recipient age. The researchers concluded that among the ML algorithms used, the Random Forest algorithm outperformed the others (sensitivity of 0.67, specificity of 0.97, positive likelihood ratio of 22.33, negative likelihood ratio of 0.34) and could potentially serve as a model for predicting severe pneumonia in KT recipients [66]. Peng et al. also assessed the risk of postoperative pneumonia, focusing on its association with immune system biomarkers. They studied the immune monitoring panel of 146 KT recipients and applied ML models for the analysis. They concluded that the significant differences in cell counts of each subpopulation between the pneumonia and no-pneumonia groups suggested that better-personalised therapy might be achieved based on immune monitoring results [67].

Besides pneumonia, cytomegalovirus (CMV) infection is considered a significant cause of post-transplant morbidity and mortality. Sheppard et al. used data from 539 KT recipients to train ANNs to identify patients at high risk of developing CMV disease. The best model built in this study achieved a sensitivity of 90% and a specificity of 83%. They concluded that the AANs outperformed clinicians in predicting CMV infection, highlighting the potential use of neural networks in KT data analysis [68].

In contrast, a study by Bae et al. reported different results compared to the majority of papers in the literature. They compared ML, gradient boosting (GB) and random forests (RF) to conventional regression in predicting KT outcomes using data from 133.431 adult Deceased-Donor KT recipients. They studied the following five KT outcomes: DGF, one-year AR, death-censored graft failure, death and all-cause graft failure. In their comparison of predictive performance, ML algorithms did not show superior discrimination over regression in any of the five aforementioned outcomes. In predicting rejection, regression (C = (0.601) 0.611 (0.621)) outperformed GB (C = (0.581) 0.591 (0.601)) and RF (C = (0.569) 0.579 (0.589)). For all other outcomes, the C-statistics were nearly identical across methods (DGF, 0.717–0.723; death-censored graft failure, 0.637–0.642; all-cause graft failure, 0.633–0.635; and death, 0.705–0.708), casting doubt on the efficacy of ML [69]. 

#### 2.3.4. Computer-Aided Diagnosis (CAD) 

Computer-aided diagnosis (CAD) refers to a set of software tools and techniques designed to assist physicians in interpreting medical images and making diagnostic decisions. CAD systems can be used to detect post-transplant complications and to assess kidney function.

In 1996, Hamilton et al. assessed post-transplant renal artery stenosis (RAS) using ANNs. They used data from 31 99mTc-MAG3 captopril renographies from hypertensive KT patients with a suspected diagnosis of RAS. Each renogram study was correlated with an arteriogram as the “gold standard”. The authors reported an accuracy of 95% for their model, suggesting almost 30 years ago that ANN analysis could be implemented as a tool to accurately interpret captopril renography investigations [70].

EL-Baz et al. proposed a new approach for the radiological classification of normal and AR allografts using dynamic contrast-enhanced Magnetic Resonance Imaging. Their three-step algorithm included isolation of the kidney from the surrounding anatomical structures, motion correction models to account for the motion of the kidney due to patient breathing, and the identification of perfusion curves showing the transportation of the contrast agent into the tissue in order to distinguish normal and AR allografts [71,72].

Finally, three papers originated from the same research group that examined the application of CAD in kidney allograft assessment.

Shehata et al. presented a CAD system for early AR detection using (3D + b-value) diffusion-weighted (DW) MRI data. The researchers used a DL-based classifier with stacked nonnegative constrained autoencoders to distinguish between rejected and nonrejected kidney transplants [73].

In 2019, Abdeltawab et al. introduced a DL-CAD system for the early detection of AR. Their proposed model represents a fusion between imaging markers, such as DW-MRI-derived parameters and clinical biomarkers, such as Creatinine clearance and serum plasma Creatinine. The imaging markers studied were the apparent diffusion coefficients (ADC) that represent the perfusion of the blood inside the transplanted kidney. The AI tool that was used was a convolutional neural network (CNN)-based classifier, which was trained with the fused data mentioned above of 56 KT recipients. The overall reported accuracy of the proposed system is 92.9%, with 93.3% sensitivity and 92.3% specificity compared to biopsy results, in distinguishing non-rejected kidney transplants from rejected ones, suggesting that the novel model could be implemented as a tool to assist physicians in assessing the post-transplant allograft status non-invasively [74].

In their third study, in 2020, they used data from 47 DW-MRI, 30 blood oxygen level-dependent MRI and the same clinical biomarkers as in the previous study. All these data were used to train a DL-based classifier to differentiate non-rejection from AR allografts to overcome the limitations of the gold standard technique, i.e., allograft biopsy. Their system demonstrated 93.3% accuracy, 90% sensitivity and 95% specificity [75].

In a different direction, a retrospective study assessed the use of computed tomography body composition analysis, using AI-based tissue segmentation, to predict patient and kidney transplant survival in elderly transplant patients. The researchers used the BMI, psoas muscle index (PMI), skeletal muscle index (SMI), visceral adipose tissue (VAT) and subcutaneous adipose tissue (SAT) as variables. They found that 1-year uncensored and censored KT survival was influenced by reduced PMI (*p* = 0.02 and *p* = 0.03, respectively) and reduced SMI (*p* = 0.01 and *p* = 0.03, respectively); 3-year uncensored KT survival was influenced by increased VAT (*p* = 0.04); and 3-year censored KT survival was influenced by reduced SMI (*p* = 0.05). Additionally, sarcopenia was found to impact 1-year uncensored KT survival (*p* = 0.05), and obesity was shown to affect 3-year and 5-year uncensored KT survival. The study concluded that AI-based body composition analysis could be valuable in predicting short- and long-term KT survival [76].

#### 2.3.5. Pathological Evaluation of the Allograft

As mentioned earlier, AI has the potential to revolutionise the histopathological examination of kidney allografts. Numerous studies have explored AI-driven digital pathology, primarily utilising ML techniques, to diagnose various allograft conditions such as AR, inflammation and chronic nephropathy. Given the extensive volume of research in this field, a comprehensive review of all studies is beyond the scope of this review. Nevertheless, several notable studies are briefly highlighted herein. A summary of relevant studies is presented in Table 1.

Most studies focus on the diagnosis of rejection. Kazi et al., in 1998, developed a Bayesian belief network based on observations from 110 transplant biopsies. Its performance was evaluated on 21 biopsies reviewed by 37 different kidney transplant pathologists. The study concluded that their model could assist pathologists in making more reliable diagnoses of early AR [77].

Three further publications of the same research group assessed the application of AI in diagnosing rejection. In their first study, they focused on T cell-mediated rejection (TCMR) and ABMR, developing the TCMR and ABMR scores using ML methods. The reported accuracies were 87% and 85%, respectively [78]. In their second study, they used ML techniques to develop additional scores for ABMR, TCMR and five related histological lesions to examine the distribution of molecular phenotypes related to rejection. They concluded that these results provide a system for precise molecular assessment of biopsies and establish a new standard for recalibrating conventional diagnostic systems [79]. Their third study aimed to optimise the accuracy and stability of their Molecular Microscope Diagnostic System (MMDx) diagnoses by replacing single ML classifiers with ensembles of diverse classifier methods. They also evaluated the use of automated report sign-outs and compared them to those generated by expert pathologists. They concluded that RF-based automated reports showed similar levels of agreement with human experts, with 92% and 94% in predicting the expert MMDx sign-outs for TCMR and ABMR, respectively [80]. 

Other studies have assessed the ability of CNNs to diagnose inflammation and chronic features in KT biopsies. Hermsen et al. used CNN to quantify healthy and sclerotic glomeruli, interstitial fibrosis, tubular atrophy and inflammation in kidney allografts. Their results showed a high correlation with conventional pathology, concluding that CNNs have the potential to improve KT diagnostics and will benefit the community as a novel method to generate surrogate endpoints for large-scale clinical studies [81]. Smith et al. employed CD45-stained slides coupled with image analysis tools to quantify the amount of non-glomerular inflammation within the cortex. They concluded that computer-assisted scoring of inflammation showed a high correlation with Banff scoring (maximum Pearson correlation 0.824), highlighting that digital image analysis provides a powerful tool for the analysis of kidney pathology [82].

**Table 1:** AI and kidney pathology studies. AR: acute rejection, CNN: conventional neural network, DL: deep learning, DC: dice coefficients, TCMR: T-cell mediated rejection, RNA-Seq: ribonucleic acid sequencing, ML: machine learning, ABMR: antibody-mediated rejection, AUROC: area under the receiver operating characteristic.

Author, Year	Objective	AI method Used	Performance
Furness [83], 1999	AR diagnosis	Neural network	19/21 correct diagnoses
Hermsen [81], 2019	Multiclass segmentation performance	CNNs/DL	DC 0.80/0.84
Liu [84], 2019	TCMR diagnosis	RNA-Seq-based ML	55/67 TCMR 65/105 ABMR
Kim [85], 2019	AR diagnosis	CNNs/DL	Sensitivity 0.7951 Specificity 0.9941
Ligabue [86], 2020	Kidney immunofluorescence reporting	CNNs/DL	Accuracy: 0.79–0.94 in different feature prediction
Wilbur [87], 2021	Glomeruli identification	CNNs	Sensitivity 86% Specificity 92%
Kers [88], 2022	Normal vs. rejection vs. other diseases	CNNs/DL	Rejection: AUROC (0.75 [0.73–0.76])
Smith [82], 2023	Non-glomerular inflammation quantification	CNNs	DC 0.858

### 2.4. Post-Transplant Care

#### 2.4.1. Immunosuppressive Therapy

Immunosuppressive treatment after KT is a complex and individualised process. It involves a combination of medications that need careful monitoring and management to prevent rejection and minimise side effects. These medications include corticosteroids, calcineurin inhibitors (Tacrolimus, Cyclosporin), mTOR inhibitors (Everolimus, Sirolimus) and antiproliferative agents (mycophenolate mofetil-MMF) [89].

##### Tacrolimus 

Tacrolimus has a narrow therapeutic window, and the dose required to achieve optimal concentrations varies considerably between patients, but even for the same patient over time. Therefore, developing individualised and data-driven immunosuppression protocols is vital to avoid graft rejection and serious adverse effects caused by inappropriate Tacrolimus levels. AI tools have applications in this domain as well [90].

Multiple studies have attempted to develop accurate and personalised models to assist physicians in tacrolimus administration. 

Back in 1991, McMichael et al. evaluated a novel ‘’intelligent’’ dosing system for optimising Tacrolimus dosing. They programmed an artificial intelligence dosing system (IDS) to predict drug doses and levels based on hundreds of dosing histories from previous patients. They developed an algorithm and then validated it in clinical practice, achieving 95% accuracy in describing the relationship between Tacrolimus dosage and plasma level [91].

Seeling et al. applied a regression tree model using KT patient data from 1995 to 2008. They developed a model to help predict Tacrolimus concentration levels, intended for implementation in clinical practice as a decision support system [92].

In 2015, a prospective study from Norway compared computerised to conventional dosing to achieve optimal Tacrolimus concentration. The researchers included 78 KT recipients and randomised them equally into the two groups. The results revealed that the proportion of concentrations within the target range was significantly higher with computerised dosing than with conventional dosing, both in standard-risk patients (medians, 90% [95% confidence interval {95% CI, 84–95%] vs. 78% [95% CI, 76–82%], respectively, *p* < 0.001) and high-risk patients (medians, 77% [95% CI, 71–80%] vs. 59% [95% CI, 40–74%], respectively, *p* = 0.04) [93]. This model could meet broad acceptance as an everyday tool to assist physicians with tacrolimus administration.

A similar study comparing the performance of multiple linear regression (MLR) and eight ML techniques in predicting Tacrolimus stable dose was conducted in a large Chinese cohort of 1045 KT patients. The ML techniques used were ANN, regression tree (RT), multivariate adaptive regression splines (MARS), boosted regression tree (BRT), support vector regression (SVR), random forest regression (RFR), lasso regression (LAR) and Bayesian additive regression trees (BART). The researchers concluded that RT performed best in both derivation [0.71 (0.67–0.76)] and validation cohorts [0.73 (0.63–0.82)], outperforming MLR, highlighting the importance of AI tools in facilitating individualised Tacrolimus administration [94].

Niel et al. programmed an ANN to predict immediate-release Tacrolimus area under the curve (AUC) in KT recipients. The algorithm was trained with 53 input parameters consisting of Tacrolimus blood concentration at three h post-dose and its corresponding AUC measure extracted by the literature. The output was the neural network AUC (nnAUC). The results revealed no significant difference between AUC and nnAUC (*p* = 0.95), making this AI tool a fast, cost-effective and clinically simple approach to estimating Tacrolimus AUC in KT recipients [95]. 

Finally, two studies that used AI tools to predict Tacrolimus concentrations explored the role of genetic polymorphisms and genotypes. Thishya et al. implemented an ANN model to predict Tacrolimus bioavailability based on the role of ABCB1 and CYP3A5 genetic polymorphisms. They identified factors such as younger age, male gender and optimal BMI, which exhibited lower bioavailability of Tacrolimus. With regards to the examined polymorphisms, ABCB1 1236 C > T and 2677G > T/A showed inverse association, while CYP3A5*3 showed a positive association with the bioavailability of Tacrolimus [96]. Cai et al. evaluated the association between Tacrolimus concentrations and endogenous CYP3A4 phenotype, CYP3A5 genotype and clinical variables in 182 KT recipients using RF algorithms. The results suggested that the endogenous CYP3A4 phenotype was the most important biomarker for predicting Tacrolimus concentrations and dose requirements, with the RF models exhibiting high goodness of fit and high predictability [97], suggesting it might be a useful tool in ameliorating post-transplant immunosuppression dosing.

##### Cyclosporin

Cyclosporin is a critical dose immunosuppressant drug with a narrow therapeutic index and significant pharmacokinetic variability, necessitating careful monitoring of blood concentrations and a personalised approach to dosing [98,99]. 

Camps-Valls et al. proposed the use of neural networks for individualising the dosage of Cyclosporine A (CyA) in patients who have undergone KT. They trained a neural network model using data from thirty-two KT patients, reaching the conclusion that it could accurately predict both the dose and blood concentrations of Cyclosporin in steady-state [100].

Similarly, Goren et al. proposed the use of the Takagi and Sugeno-type “adaptive-network-based fuzzy inference system” (ANFIS) to predict the concentration of CyA in blood samples from KT patients. They developed their model using data from 138 KT patients and 20 input parameters, concluding that it can serve as a decision support system to assist physicians in determining the optimal therapeutic drug dose in clinical settings [101].

##### MMF 

Dosing for MMF and its active form (mycophenolic acid-MPA) is less studied compared to Tacrolimus. Although MMF was originally marketed as a fixed-dose agent, a recent international consensus report advocated a personalised approach to its dosing by estimating the MPA AUC [102]. In 2021, Woillard et al. published a study on predicting MPA therapeutic drug monitoring using ML-techniques [103]. Their model was trained on 12,877 MPA AUC values from 0 to 12 h requests, collected from 6884 transplant patients. They developed two ML models based on two or three concentrations of MPA measured at least at three sampling times (20 min, 1 and 3 h after dosing). Their ML models performed better than maximum a posteriori (MAP) Bayesian estimation in four independent full-pharmacokinetic datasets, leading the authors to conclude that they can be used for routine exposure estimation and dose adjustment [103].

An interactive tool, the CISTEM Immunosuppression Complication Risk Rejection Tool, has been made available online for predicting complications based on immunosuppression, utilising data from both donors and recipients [104].

#### 2.4.2. Dietary Issues 

A carefully managed diet can be vital for the long-term success of a KT and overall health. Previous studies have shown that adherence to the Mediterranean diet (MD), which consists of a high intake of fish, fruit, vegetables and olive oil, combined with a low intake of dairy and red meat products, is associated with improved kidney function [105,106]. In KT recipients, following the MD is associated with better post-transplant kidney function outcomes and a reduced risk of new-onset diabetes after transplantation [107,108]. AI tools have been used to identify the effect of specific diets on selected blood parameters of KT patients. Researchers used ANNs to study the impact of the MD in lipid parameters post-transplant. They concluded that the MD could benefit KT recipients without serious dyslipidaemia, but those with substantial dyslipidaemia should receive appropriate medication treatment combined with MD [109]. This demonstrates that AI tools can be used to identify which patients will benefit from a specific intervention and improve their post-transplant care.

#### 2.4.3. Medical Wearables 

Medical wearables monitoring vital signs, such as heart rate, temperature, blood pressure, oxygen saturation and physical activity, can be utilised to help and support patients remotely. Recently, these devices have started to incorporate AI tools to improve their efficacy [110]. The use of these devices in post-transplant care is still in its infancy [6,111]. With these devices, patients can be monitored remotely, reducing the number of hospital visits and the costs of potential hospitalisations, offering convenience and comfort for patients and their families [7]. Furthermore, these wearables can collect vast amounts of data that can be used to train new ML models in pursuit of providing better patient care. The most promising application of AI-associated medical wearables in CKD and KT patients is the use of DL techniques to analyse electrocardiogram (ECG) patterns on smartwatches to detect high potassium levels in the blood [7].

#### 2.4.4. Prediction of Long-Term Outcomes

Predicting long-term outcomes in KT patients is vital for improving patient survival and quality of life, optimising the use of the limited pool of donor organs, reducing healthcare costs, enhancing clinical decision-making, facilitating long-term care planning, advancing research and ultimately improving overall transplant success rates. AI has various applications in this domain as well, mainly owing to its ability to analyse vast amounts of clinical data, leading to more accurate predictions.

The first study using AI tools to identify variables associated with chronic allograft rejection dates back to 1999. The authors used an ANN to select the most important parameters correlating with chronic rejection (CR) progression. They managed to reduce the initial 33 studied variables to 8, which showed a strong influence on CR progression [112].

Lofaro et al. proposed an identification model based on ML to predict chronic allograft nephropathy (CAN). The researchers analysed 80 KT patients with a 60-month follow-up and divided them into two classes (CAN vs. no CAN). Serum creatinine, estimated glomerular filtration rate (GFR) with Modification of Diet in Renal Disease study formula (MDRD), serum haemoglobin, haematocrit, blood urea nitrogen and 24-h urine protein excretion were the variables associated with CAN. The first model selected six predictors of CAN, showing a sensitivity of 62.5%, a false-positive rate of 7.2%, and an AUC of 0.847 (CI 0.749–0.945). The second model selected four variables, showing a sensitivity of 81.3%, a false-positive rate of 25%, and an AUC of 0.824 (CI 0.713–0.934). The above led to the conclusion that the use of classification trees represents a valid alternative to traditional statistical models [113].

Badrouchi et al. developed an ML model to predict long-term outcomes of KT (in terms of long-term survival, graft function at five years or more and return to dialysis) based on ten variables. These variables included, in decreasing order of importance, the following: hypertension, history of red-blood-cell transfusion, early acute kidney injury post-KT, early AR, CMV infection, length of first hospitalisation, MMF therapy, donor’s age, three-month estimated GFR and time on dialysis before KT. They included 407 KTs and divided them into two groups (group A, with a graft lifespan greater than five years and group B, with poor graft survival). Among the 35 AI models developed, the best model had an AUC of 89.7% (Sensitivity: 91.9%; Specificity: 87.5%). The authors concluded that their model can be used as a decision-support tool to early prognosticate graft status [114].

Another study evaluating post-transplant chronic kidney failure implemented AI tools to develop a clinical decision support system (CDSS). The researchers proposed a fuzzy logic-based CDSS for the follow-up of KT patients, assessing two parameters: proteinuria and GFR. These important values are associated with various stages of kidney failure, allowing the characterisation of the severity of the condition. The researchers reported an accuracy greater than 90% for their system, concluding that their CDSS is applicable to clinical practice, providing effective support to clinicians [115].

In a large international, multicohort study including 13.608 KT recipients, researchers developed DIPSO, a dynamic, integrative system for predicting outcomes. They created deeply phenotyped cohorts of transplant recipients, incorporating various data: clinical, histological, immunological variables and repeated measurements of eGFR and proteinuria to assess long-term allograft survival. Their Bayesian model demonstrated high prediction performance (overall dynamic AUC 0.857 [95% CI 0.847–0.866]) and was validated on a large scale, making it a potential tool for decision-making and guiding clinicians in managing KT recipients [116].

## 3. Future Applications of AI in KT

The future applications of AI in KT hold immense promise, potentially transforming every stage of the process. The integration of AI into electronic patient registration systems will streamline the management of patient information, making it easier to track and analyse patient data in real time. AI-powered surveillance systems, expanding existing databases, can monitor patient outcomes and detect early signs of complications. By creating an international collaborative network through data sharing and research outputs, collective knowledge and best practices worldwide can be significantly enhanced, leading to improved transplant success rates [117]. To fully exploit these benefits, guidelines need to be developed to support the use of AI in organ allocation and the prediction of rejection.

AI may also play a significant role in advancing regenerative medicine and bioengineering techniques, including organ printing and the development of bioartificial kidneys. AI tools can analyse large datasets to identify patients who may benefit from regenerative therapies, optimise treatment plans and refine materials and fabrication methods for tissue engineering applications [118]. In the future, AI could revolutionise the machine perfusion process as well. Through personalised perfusion protocols and customised settings tailored to the specific needs of each kidney, AI can optimise preservation effectiveness, maintain the organ’s health and improve its chances of successful transplantation. A potential future application could also involve the development of automated AI-powered monitoring systems that could provide continuous oversight of the machine perfusion process. These systems could detect deviations from optimal conditions and alert medical staff to intervene promptly, ensuring the organ remains in the best possible condition. Furthermore, in the near future, AI is expected to enhance its role in the surgical process as well through novel emerging AI robotic surgery platforms [119].

## 4. Limitations 

AI in medicine holds significant promise, yet it also has notable limitations. Since ML is trained through data collection and processing to make predictions, the training process requires long periods of time and a vast amount of comprehensive and high-quality data [6]. Furthermore, before an AI algorithm can be implemented in clinical practice, it is essential to validate it in real-world studies [7]. Bias and suboptimal quality of training data can lead to inaccurate predictions or recommendations, disproportionately affecting certain populations. It should always be kept in mind that a mistake in the algorithm, compared to a single doctor’s mistake, can cause major harm to numerous patients.

Another important limitation of AI is the lack of interpretability. The “black box” nature of many AI algorithms makes it challenging to interpret and trust their decisions, which is crucial in medical settings where understanding the rationale behind a recommendation is essential [6]. Although some scientists compare the black box of AI to the ‘’human black box’’, the brain, stating that likewise its functioning remains more or less unknown, many others view AI as a tool to be used with caution [120]. The preservation of patient privacy and the security of data is another important issue regarding AI. Hacking, security breaches and intents of algorithm manipulation are serious risks that may limit the application of AI in medicine. Furthermore, the incorporation of AI-related private-sector investments and designers poses the potential danger of manipulating the clinical decision-support systems to serve specific financial interests without necessarily optimising healthcare [117,121]. In addition to security concerns, AI in healthcare also raises a number of ethical issues. Since AI systems learn from the data they are trained on, if the training data are biased, AI algorithms can inadvertently perpetuate biases in healthcare data, leading to disparities and inaccurate outcomes [122]. Additionally, when an AI system makes a mistake, it’s often unclear who is responsible—the AI developer, the healthcare provider, or the hospital that deployed the system. This creates legal and ethical challenges regarding accountability [123].

Finally, the wide implementation of AI into clinical practice may cause potential negative effects on the nature of the relationship between patients and physicians and could also lead to the weakening of the clinicians’ decision-making abilities. If AI tools became too prevalent in the future, patients might not necessarily have an attending physician, and younger doctors might less well develop the skills of communication with patients, performing differential diagnoses, and making informed decisions for their patients.

## 5. Conclusions

In conclusion, the use of AI in KT holds tremendous potential for revolutionising patient care. AI is, without a doubt, an integral part of the future of medicine, offering powerful tools to enhance personalised approaches and improve outcomes. However, it is essential to recognise that AI is not here to replace human expertise but to work alongside healthcare professionals, augmenting their capabilities and providing valuable insights. As we move towards more individualised treatment plans, AI can help tailor strategies to each patient’s unique needs, ensuring more precise and effective care. Nonetheless, more research is needed to address the current limitations and ensure the safe and ethical integration of AI into clinical practice. By fostering collaboration between AI technologies and medical practitioners, we can pave the way for a future where advanced, personalised care becomes the standard in KT and beyond.

## Data Availability

Data sharing is not applicable.
